# Which dressing do donor site wounds need?: study protocol for a randomized controlled trial

**DOI:** 10.1186/1745-6215-12-229

**Published:** 2011-10-17

**Authors:** Anne M Eskes, Fleur E Brölmann, Louise AA Gerbens, Dirk T Ubbink, Hester Vermeulen

**Affiliations:** 1 Quality Assurance & Process Innovation, Academic Medical Center, University of Amsterdam, Amsterdam, the Netherlands; 2 Amsterdam School of Health Professions, Amsterdam, the Netherlands; 3 Surgery, Academic Medical Center, University of Amsterdam, Amsterdam, the Netherlands; 4 Amsterdam School of Health Professions, Amsterdam, the Netherlands

## Abstract

**Background:**

Donor site wounds after split-skin grafting are rather 'standard' wounds. At present, lots of dressings and topical agents for donor site wounds are commercially available. This causes large variation in the local care of these wounds, while the optimum 'standard' dressing for local wound care is unclear. This protocol describes a trial in which we investigate the effectiveness of various treatment options for these donor site wounds.

**Methods:**

A 14-center, six-armed randomized clinical trial is being carried out in the Netherlands. An a-priori power analysis and an anticipated dropout rate of 15% indicates that 50 patients per group are necessary, totaling 300 patients, to be able to detect a 25% quicker mean time to complete wound healing. Randomization has been computerized to ensure allocation concealment. Adult patients who need a split-skin grafting operation for any reason, leaving a donor site wound of at least 10 cm^2 ^are included and receive one of the following dressings: hydrocolloid, alginate, film, hydrofiber, silicone dressing, or paraffin gauze. No combinations of products from other intervention groups in this trial are allowed. Optimum application and changes of these dressings are pursued according to the protocol as supplied by the dressing manufacturers. Primary outcomes are days to complete wound healing and pain (using a Visual Analogue Scale). Secondary outcomes are adverse effects, scarring, patient satisfaction, and costs. Outcome assessors unaware of the treatment allocation will assess whether or not an outcome has occurred. Results will be analyzed according to the intention to treat principle. The first patient was randomized October 1, 2009.

**Discussion:**

This study will provide comprehensive data on the effectiveness of different treatment options for donor site wounds. The dressing(s) that will prevail in effectiveness, satisfaction and costs will be promoted among clinicians dealing with such patients. Thus, we aim to contribute a well-designed trial, relevant to all clinicians involved in the care for donor site wounds, which will help enhance uniformity and quality of care for these patients.

**Trial registration:**

http://www.trialregister.nl, NTR1849. Date registered: June 9, 2009

## Background

Split skin grafting (SSG) is a widely used reconstructive technique to repair skin defects (e.g. burns, chronic, and traumatic wounds) [1,2], including those that cannot be covered by a skin flap or are not likely to heal by secondary intention [3]. The wound created after harvesting the skin is called the donor site wound (DSW). Depending on the thickness of the SSG, the DSW should re-epithelialize completely in 7 to 21 days [3]. Optimum local care for these DSWs should promote wound healing and be cost-effective, while it should prevent complications, such as pain, discomfort, infection, and scarring. Particularly pain and discomfort are reported to occur more frequently from DSWs than at the recipient site [3-5].

Clinical practice shows a large number of dressings and topical agents for DSWs, while the optimum dressing choice for local wound care is unclear [1,2,6,7]. Consequently, large variation exists among health care professionals regarding their choice for wound dressing materials or topical agents to treat DSWs [8-10]. Based on national surveys, alginates appear to be the most commonly used primary dressing [8-10], probably due to their additional haemostatic properties [11,12]. They are followed by films, hydrofibers, silicone dressings and paraffin gauzes [8].

Available evidence comprises four systematic reviews (SRs), presenting a lack of strong evidence for the effectiveness of the different dressings for the treatment of DSWs, especially for alginates [1,2,6,7]. These SRs tentatively conclude that moist dressings are preferable over non-moist dressings in terms of wound healing. Hydrocolloid and films seem better than nearly all other materials (e.g. alginates, paraffin gauzes, hydrofibers, and foams) as to healing and pain [7]. Hydrofibers in turn seem to outperform tulle dressings in terms of wound healing and pain [13,14]. Although tulle dressings seem to be least suitable for the local treatment of DSWs, recent evidence shows that gauze-based dressings still have a place in wound care [15]. Some centers still adhere, or have returned to, these gauze-based dressings [16]. Silicone-based dressings have the advantage of being non-adhesive, although they tend to dislocate easily and do not seem to outperform alginates [17]. These conclusions are formulated cautiously as most authors state that more well designed and rigorous studies are needed.

We therefore conceived a trial to compare the six most promising dressing groups, based on common usage and available evidence. In this paper we will report on the design of our 14-center six-armed randomized clinical trial (RCT). This trial received the acronym "Rembrandt" trial, which stands for Recognizing Effective Materials By Randomizing & Assessing New Donorsite Treatments. In this trial we aim to answer the following question: Which of the following dressing materials for DSW of SSGs stand out in effectiveness: hydrocolloids, alginates, films, hydrofibers, silicone dressings, or paraffin gauzes, in terms of wound healing, adverse effects (e.g. pain and scarring), and costs?

## Methods

### Protocol and registration

The methods applied in our 14-center RCT were specified in advance, documented in a protocol, and registered (http://www.trialregister.nl, NTR 1849). The study was approved by the local medical ethics committee and by the institutional review boards of each participating hospital or burns centre. The methods used are summarized here according to the revised CONSORT Statement [18].

### Design and setting

**Figure 1:**
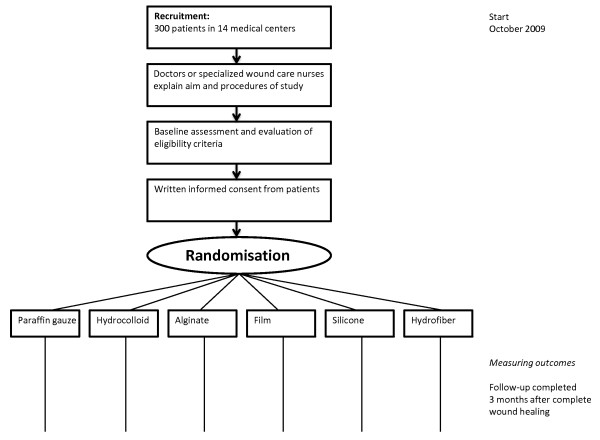
**Flowchart of the Rembrandt Trial**.

We designed a national, 14-center RCT with six treatment groups in the Netherlands (Figure 1). The coordinating center (Academic Medical Center at the University of Amsterdam) invited hospitals (i.e. departments of surgery, plastic surgery and otorhinolaryngology) and burns centers to participate in the trial, resulting in 13 contributing hospitals (4 university hospitals, 5 teaching clinics and 4 general hospitals) and 1 burns center.

### Eligibility criteria for patients

In this trial we include all adult patients, either hospitalized or under treatment in the outpatient clinic in one of the contributing hospitals or burns centre, who need a SSG-operation for any reason. The DSW should have a minimum size of 10 cm^2 ^(to allow proper application and investigation of the study dressings) and be suitable for all treatment options in the trial. Patients are included after full, understandable and neutral explanation by the treating physician or coordinating investigators and after giving written informed consent. Patients are excluded when they receive a treatment known to seriously impair normal wound healing (e.g. chemotherapy, corticosteroids, or local irradiation therapy) or if patients are not physically or mentally able to consent.

### Interventions

Before starting the trial, manufactures of the products involved were invited to develop a protocol how best to apply their wound materials for DSWs, to ensure correct and uniform application of the six different dressing groups:

1. A paraffin gauze-based material (e.g. Jelonet^®^, Adaptic^®^);

2. A hydrocolloid (e.g. DuoDERM^® ^E);

3. An alginate (e.g. Kaltostat^®^, Algisite^®^, Melgisorb^®^);

4. A semi-permeable film (e.g. Tegaderm^®^, Opsite^®^);

5. A silicone dressing (e.g. Mepitel^®^);

6. A hydrofiber (e.g. Aquacel^®^).

The coordinating investigators (FEB and AME) or dressing manufactures orally instructed medical and nursing staff on the wards and out-patient clinics of the contributing centers at the beginning of the trial. Furthermore, they received written application advices as reminders for a uniform treatment protocol, e.g. regarding change frequency and treatment duration. Posters and pocket charts were also distributed to inform about inclusion criteria and contact persons.

#### Surgical procedure

A SSG operation is to be performed with an electric or pneumatic dermatome or free hand-knife, according to local best practice. The SSG should preferably be between 0.20 and 0.30 mm to achieve a reasonably uniform depth of the DSW and should be taken from thighs, arms or buttocks. Method of hemostasis of the DSW is at the discretion of the surgeon (e.g. adrenaline-soaked gauze). However, the decision to use hemostasis must be made before randomization and will be recorded.

#### Wound treatment

Local wound care according to the assigned dressing group starts directly after randomization (see heading Randomization). The brand of the dressing will be recorded. No combinations of products from other dressing groups in this trial are allowed to ensure that the effect found after completion of the trial can be attributed only to the dressing to which the patient was allocated. The optimum changing frequency will be pursued as advised for each dressing material. This may differ from no dressing changes (e.g. hydrofiber) to daily changes in case of leakage (e.g. hydrocolloid or paraffin gauze). We allow coverage of the primary dressings with cotton gauzes and bandages. The type of secondary dressings used will be recorded. Furthermore, we will record any crossovers and make sure the patient returns to the initially allotted dressing.

#### Co-interventions

Additional wound debridement, cleansing or protection may be indicated during a dressing change and is allowed in all treatment arms, as this reflects real life. In case of an (impending) wound infection, the wound may be treated with iodine (Povidone- or Cadexomer iodine), to be applied beneath the allotted dressing material [19]. The type of iodine used will be recorded.

### Study outcomes

#### Primary endpoints

The primary endpoint with respect to the effectiveness of wound dressings in the treatment of DSWs is time to complete wound healing. We define wound healing as re-epithelialization of the total wound surface. We decided this is not the case until all crusts have come off. This endpoint is to be assessed by an independent investigator who is not aware of the treatment given. The second primary outcome is pain from the donor site area. It is documented by the patient on a Visual Analogue Scale (VAS), varying from 0 (no pain) to 10 (intolerable pain). This is scored daily for the first two weeks postoperatively and twice a week during the third and fourth week, in a patient-held diary. Both primary endpoints are meaningful and relevant to patients and were therefore used for the sample size calculation [20].

#### Secondary endpoints

As secondary endpoints we assess the occurrence of local complications, e.g. wound infections, based on clinical symptoms of infection, scarring at 12 weeks postoperatively (using Patient and Observer Scar Assessment Scale (POSAS) assessed by the patients themselves and researchers, treating physicians or specialized wound nurses) [21], patient satisfaction (varying from 1 (absolutely dissatisfied) to 10 (absolutely satisfied), and costs (material and nursing costs). Itching scores are also collected by using a VAS, ranging from 0 (no itching) to 10 (intolerable itching) and obtained through the patient-held diary.

### Randomization

Patients are to be randomized in the operation theatre, just after the skin harvest and hemostasis, and before the DSW is to be dressed. In each contributing center an appointed officer performs the randomization using an online computer software program (ALEA NKI-AVL, Amsterdam, The Netherlands, Release: 2.2.) to ensure allocation concealment. The trial is stratified by center, with a balanced allocation ratio for each treatment arm using a biased coin [22]. The biased coin method preserves most of the unpredictability associated with simple randomization [22].

### Blinding

Blinding of patients and care-providers (e.g. doctors and nurses) is not possible because the treatment options cannot be masked. To overcome this possible source of performance bias, independent doctors and nurses of the outpatient clinic, who are unaware of the treatment allocation, will assess whether or not an endpoint has occurred [23].

### Sample size

The study size calculation (using nQuery Advisor version 7.0, Statistical Solutions Ltd, Cork, Ireland) was based on two primary outcomes using a one-way analysis of variance. To detect a 25% quicker mean wound healing, which is in agreement with the study of Wiechula [2], and with a 5% significance level, a power of 90%, and a standard deviation (SD) of 3 days, a sample size of 50 patients per group is necessary, given an anticipated dropout rate of 15%. This number also allows detection of a minimum difference in pain scores of 2.0 or greater (with a SD of 2) on the VAS and discernment of a cost difference of €2 per day. To recruit this number of patients an 18-month inclusion period is anticipated based on the performed number of SSGs as estimated by each of the 14 co-operating hospitals.

### Data collection

The coordination center designed a standardized case record form (CRF) and distributes this in a paper-based or electronic version. The latter one is made available through a secured website, http://www.rembrandt-trial.nl (using Joomla, an open-source website software package), which also facilitates remote patient randomization and data entry. We collect copies of all completed forms from the co-operating hospitals and maintain the database using SPSS software (PASW statistics version 18.0, IBM, Armonk, NY, USA). Data are collected on baseline demographic and clinical patient characteristics of each group, whether the patients receive the allotted treatment and complete the study protocol, and are analyzed for the primary outcomes. Patients whose treatment deviates from the initial allocation will be described together with the reasons for this. Data on all important adverse events or side effects in each intervention group are recorded as well. Data from the patient-held diaries will be returned to the coordinating center. Double data entry will be conducted by FEB and AME, and compared using the SPSS Data Entry Builder program (Release 4.0.2). We will resolve any discrepancies by discussion and by re-checking the data.

### Data monitoring

Data completeness is reviewed weekly, and reminders or queries are sent timely. FEB and AME visit the cooperating hospitals on a regular basis to promote the trial and to be closely associated with the data collection. Thus, an accurate and complete data set is ensured. Because no (serious) adverse effects were expected from the commercially available dressings that would require interim analysis, we refrained from installing a data safety monitoring board.

### Data analysis

Data coding and analysis will be carried out using SPSS software (PASW statistics version 18.0, IBM, Armonk, NY, USA). Differences in outcome variables will be analyzed on an intention-to-treat basis. A general linear model will be used to analyze the differences between the treatment arms for the various endpoints measured repeatedly, as the data are likely to be unequally distributed. Differences in wound healing time between the dressing groups will be examined using the Kaplan-Meier method and the Mantel-Cox log-rank test. Data analysis will be conducted by the authors and replicated by the Clinical Research Unit of the coordinating hospital. We will record any crossovers and make sure the patient returns to the initially allotted dressing. Missing data are dealt with by using the Generalized Estimating Equations model in our statistical analysis.

### Data storage

Data are stored at the coordinating center in a Trial Master File and at the co-operating hospital sites, where an Investigator File is kept. After finishing the trial, data will be saved for at least 5 years, in accordance with the recommendations as to low risk studies of the Dutch Federation of University Medical Centers.

## Discussion

In current clinical practice, a 'standard' wound such as a DSW does not appear to be associated with a uniform dressing choice [8]. Also the systematic reviews of available literature report a large clinical heterogeneity among the available trials [1,2,6,7]. To date, available evidence allows the tentative conclusion that dressings creating a moist environment seem to be preferable over gauze-based dressings in the management of DSWs. This recommendation is tentatively formulated because strong recommendations for clinical practice are hard to draw, mainly due to the poor quality and small sample sizes of the available trials. Therefore, most systematic reviews recommend new and large randomized clinical trials [2,6,7].

However, doing research in the realm of wound care involves much more than simply comparing wound care products in eligible patients. It poses many methodological and practical challenges in the design and execution of trials [24].

The first challenge is the design of our, intentionally pragmatic, RCT. To enhance the applicability and generalizability of the results of this trial, we chose a multicenter trial design and recruited patients from low- and high-volume centers like teaching hospitals and burns centers. We realize that for many surgical procedures, patients have better outcomes in high-volume centers [25-29]. However, split-skin grafting is a rather common procedure, also in smaller hospitals. Second, we are forbearing regarding local clinical care, for example by allowing several brands within each dressing group, different depths of skin grafting, and different methods of hemostasis. This helps mimicking 'real life', at the cost of losing some contrast between the six treatment arms.

Although we are liberal and pragmatic at some points, we feel we need to and can be strict in others. We urge the contributing centers to adhere to the same dressing type until complete wound healing is reached. This allows us to appreciate the true effects of each of the dressing types studied. Some argue this is not reflecting common practice [30], in which the dressing type is changed in response to any change in the clinical condition of the wound during the healing process. We do not think this will be a frequently occurring issue since these superficial DSWs usually have fairly short healing times. The protocol does allow for an antiseptic agent to be added in case of an (impending) wound infection.

Another frequent methodological challenge in wound care research is the use of subjective or surrogate outcome variables. In this trial we aim to measure our endpoints in a reliable and valid way. We strictly predefined our primary endpoint, time to complete wound healing. In a previous study it was shown that, by using this strict definition, specialized nurses had a better inter-observer agreement than doctors or nurses regarding the assessment of complete wound healing [31]. Therefore, in this trial predominantly specialized nurses will assess our primary endpoint. Our second primary outcome variable, pain, is measured using VAS scores. This is a reliable and acknowledged scale for general clinical use [32,33].

Today, financial support is a necessity to properly conduct a (multicenter) trial. For this purpose, we obtained funding from an independent institution, i.e. the Dutch Burns Foundation, which is to be preferred over subvention from one or more dressing manufacturers to avoid any publication bias. To avoid any conflict of interest, analysis and reporting of the trial stays the domain of the investigators.

The strengths of this trial are firstly the fruitful collaboration with manufacturers, who developed a dedicated protocol for the treatment of the DSW with their product. This greatly supports the uniform application of each dressing type under study. Second, using this six-armed, multicenter trial we investigate the effectiveness of the dressings most commonly used in the Netherlands and most promising from the available literature. This will facilitate implementation of the results. We expect to present the results of this trial in the course of 2012.

## Abbreviations

CRF: case-record form; CRU: Clinical Research Unit; CONSORT: Consolidated Standards of Reporting Trials; DSW: donor site wound(s); POSAS: Patient and Observer Scar Assessment Scale; RCT: randomized clinical trial; SD: standard deviation; SR: systematic review; SSG: split skin graft(s); VAS: Visual Analogue Scale.

## Competing interests

The authors declare that they have no competing interests.

## Authors' contributions

AME participated in the trial design and drafted the manuscript. FEB participated in the trial design and was involved in drafting the manuscript. LAAG has made substantial contributions to the design of the trial and gave her intellectual input on the manuscript. DTU and HV were involved in designing of the trial, obtained funding for the trial, and gave intellectual input on the manuscript. They are the project leaders and have overall responsibility for the trial. All authors have read and approved the final version of the manuscript.

## Financial support

Unrestricted grant from the Dutch Burns Foundation. They have no say in the analysis and publication of the results of this trial.

## The Rembrandt study group

Academic Medical Center: J.C. Goslings; Martini Hospital Groningen: S.J.M. Jongen; Leiden University Medical Center: J.F.A. van der Werff; operating at that time at University Medical Center Utrecht: A.H. Schuurman; Free University Hospital Amsterdam: F.B. Niessen; OLVG Amsterdam: A.C. Vahl; Kennemer Gasthuis: A.K.J. Ahmed; Spaarne Hospital: D. Nio; Tergooi Hospitals: N. Koedam; Waterland Hospital: P. Heres; Isala Klinieken Zwolle: E.G.J.M. Pierik; Rijnland Hospital; J.F.A. van der Werff; operating at that time at Gelre Hospitals Zutphen; M.L.M.J. Goessens; Lange Land Hospital: L. Levert-Brand.
